# Respiratory and Cardiovascular Health Effects of e-Cigarette Substitution: Protocol for Two Living Systematic Reviews

**DOI:** 10.2196/29084

**Published:** 2021-05-27

**Authors:** Renee O'Leary, Maria Ahmed Qureshi, Giusy Rita Maria La Rosa, Robin W M Vernooij, Damian Chukwu Odimegwu, Gaetano Bertino, Riccardo Polosa

**Affiliations:** 1 Center for the Acceleration of Harm Reduction University of Catania Catania Italy; 2 Department of Clinical and Experimental Medicine University of Catania Catania Italy

**Keywords:** cardiovascular, e-cigarettes, ENDS, respiratory, tobacco harm reduction

## Abstract

**Background:**

Despite the clear risks of tobacco use, millions of people continue to smoke. Electronic nicotine delivery systems (ENDS), commonly called e-cigarettes, have been proposed as a substitute for those who are unwilling or unable to quit. Current systematic and narrative reviews on the health effects of ENDS use, particularly respiratory and cardiovascular effects, have come to differing conclusions.

**Objective:**

We conducted two systematic reviews to critically assess and synthesize available human studies on the respiratory and cardiovascular health effects of ENDS substitution for people who smoke. The primary goal is to provide clinicians with evidence on the health effects of ENDS substitution to inform their treatment recommendations and plans. The twin goal of the reviews is to promote health literacy in ENDS users with facts on the health effects of ENDS.

**Methods:**

These two reviews will be living systematic reviews. The systematic reviews will be initiated through a baseline review. Studies will be evaluated using the JBI quality assessment tools and a checklist of biases drawn from the Centre for Evidence Based Medicine Catalogue of Bias. A narrative synthesis is planned because of the heterogeneity of data. A search for recently published studies will be conducted every 3 months, and an updated review will be published every 6 months for the duration of the project or possibly longer.

**Results:**

The baseline and updated reviews will be published in a peer-reviewed journal. The findings of the reviews will be reported in a white paper for clinicians and a fact sheet for people who use ENDS.

**Conclusions:**

The substitution of ENDS for cigarettes is one way to potentially reduce the risks of smoking. Clinicians and their patients need to understand the potential benefits and possible risks of substituting ENDS for cigarettes. Our living systematic reviews seek to highlight the best and most up-to-date evidence in this highly contentious and fast-moving field of research.

**Trial Registration:**

PROSPERO CRD42021239094; https://www.crd.york.ac.uk/prospero/display_record.php?RecordID=239094

**International Registered Report Identifier (IRRID):**

DERR1-10.2196/29084

## Introduction

### Background

There are 1.3 billion people worldwide who use tobacco, and more than 7 million of them die annually from its use [1]. Up to 11.5% of the global mortality can be linked to smoking [2]. For respiratory diseases, smoking is the attributable mortality risk factor for 64.21% of lung, tracheal, and bronchial cancer; 63.44% of laryngeal cancer; 48.47% of chronic obstructive pulmonary disease; 15.52% of tuberculosis; 11.93% of asthma; and 11.04% of lower respiratory infections [3]. For cardiovascular diseases, smoking was the attributable risk factor for 34.6% of deaths from aortic aneurysm, 26.8% from peripheral artery disease, 18.41% from ischemic heart disease, and 14.2% from stroke [4]. Smoking is one of the primary acquired risk factors for atherosclerotic disease [4].

Despite the clear risks of tobacco use, millions of people continue to smoke. Smoking has pleasurable effects [5], and some smoke for emotional regulation or to self-medicate their symptoms of schizophrenia or Parkinson disease [6]. For those who want to quit, the success rate for cessation attempts is low—approximately 7% at 6 months [7,8]. Furthermore, presently around 70% of the world’s population has no access to appropriate tobacco cessation services [9]. Quitting smoking is difficult, many have no support to quit, and some people do not wish to quit.

Electronic nicotine delivery systems (ENDS), commonly called e-cigarettes, have been proposed as a substitute for those who are unwilling or unable to quit [10,11]. A review by the US National Academies of Sciences, Engineering, and Medicine (NASEM) [12] states the following: “There is substantial evidence that, except for nicotine, under typical conditions of use, exposure to potentially toxic substances from e-cigarettes is significantly lower compared with combustible tobacco cigarettes.” The acceptability of ENDS among people who smoke is demonstrated by its rapid uptake; in 2018, there were 41 million people using ENDS compared with 7 million users in 2011 [13]. Clinicians want to know the health effects of ENDS use, asking “are e-cigarettes marginally safer, thus still too risky to substitute for combustible products, or are they substantially safer?” [14].

### Prior Reviews: Respiratory Effects

There have been 3 recent systematic reviews published on the respiratory effects of ENDS. The study by Bals et al [15], which was prepared for the European Respiratory Society, is based on studies published through August 2016, rendering it out-of-date. The review by Wang et al [16] exclusively included in vitro and in vivo studies but not human studies. Goniewicz et al [17] analyzed only cross-sectional studies of risk. Although they found a 40% reduction in adverse pulmonary outcomes, cross-sectional studies can only demonstrate an association not causation.

In addition, 4 narrative reviews on the pulmonary effects of ENDS have been published since 2019; they were conducted with a combination of in vitro, in vivo, emission toxicology, and human studies. The reviewers came to diametrically opposed conclusions regarding the respiratory effects. Gotts et al [18] and Miyashita and Foley [19] concluded that there is sufficient evidence of respiratory harm from ENDS use. In contrast, Traboulsi et al [20] presented evidence of both beneficial and adverse effects for people who smoked, whereas Polosa et al ([21] disclosure: RP and RO are coauthors) argued that ENDS substitution resulted in primarily beneficial health effects.

### Prior Reviews: Cardiovascular Effects

Recent systematic reviews offer substantially different conclusions on the cardiovascular effects of ENDS, some suggesting harm and others not finding harm, whereas still others stating that there is a lack of evidence. One systematic review of in vitro, animal, and human studies presented the conclusion that “most studies suggest potential for cardiovascular harm” caused by sympathetic nerve activation, oxidative stress, endothelial dysfunction, and platelet activation [22]. Another systematic review found no indications of a significant increase or reduction in cardiovascular disease outcomes (stroke, myocardial infarction, and coronary heart disease) among former smokers who transitioned to ENDS based on cross-sectional population studies of ENDS users [17]. Benowitz and Fraiman [23] concluded that ENDS use is likely to be associated with lower cardiovascular risks than cigarette smoking, based on toxicity studies, known mechanisms, and laboratory models. Two current systematic reviews of human studies on the cardiovascular effects of ENDS displayed more agreement on study evidence, finding lower acute effects and no chronic increases in heart rate and blood pressure for ENDS use compared with smoking [24,25].

Conversely, some reviewers claim that evidence is lacking or insufficient. The NASEM stated that “there is no available evidence whether or not e-cigarette use is associated with clinical cardiovascular outcomes (coronary heart disease, stroke, and peripheral artery disease) and subclinical atherosclerosis (carotid intima-media thickness and coronary artery calcifications)” [12]. D’Amario et al [26] stated that there is a lack of clear or conclusive data on ENDS use and cardiovascular health. MacDonald and Middlekauff [27] concluded their narrative review of human and emission toxicology studies by stating that “the effects of ECs on long-term cardiovascular health are inconclusive, but concerning.” Buchanan et al [28] in their narrative review of preclinical and clinical studies even dismiss the available literature: “While the current but still limited literature suggests that e-cigarette use may lead to fewer negative cardiovascular effects than conventional cigarettes, our review supports that there is not sufficient data to conclusively make these resolutions.”

An umbrella review [29] (review of reviews) included 7 systematic reviews but did not include the NASEM review. The umbrella review included 3 reviews conducted in 2016 or earlier when substantially fewer studies had been published and 1 review that contributed only 2 case studies from cannabinoid ENDS use. The remaining 3 systematic reviews are discussed in the previous paragraphs. The reviewers suggest that ENDS may provide a strategy for harm reduction with the caveat of the need for more studies.

### Research Question

Ascertaining the real-life effects of ENDS substitution on respiratory and cardiovascular health is complex. The complexity is because of the frequent use of ENDS in combination with conventional cigarettes, the differences in ENDS products, variations in the nicotine concentration of liquids, and the varying levels of daily exposure. There is an urgent need for a current systematic review of research, given the disagreements among prior reviews. Many of the available reviews have limitations arising from their reliance on nonhuman study data. Furthermore, several reviews have shown evidence of biased reporting. In addition, findings from recently published studies not covered in these reviews may render their conclusions obsolete. By conducting 2 living systematic reviews, we aim to answer the question: “What are the respiratory and cardiovascular health effects resulting from the substitution of ENDS for conventional cigarettes?”

### Population, Intervention, Comparator, and Outcomes Criteria

The following summarize the population, intervention, comparator, and outcomes criteria:

- Population: adults who smoke cigarettes.
- Intervention: substitution of ENDS for cigarettes.
- Comparator: participants who continue to smoke, baseline changes in respiratory or cardiovascular tests of study participants who substitute ENDS for smoking (within-subject), or comparisons with documented smoking outcomes.
- Outcomes (respiratory): changes in chronic cough, phlegm, wheezing, dyspnea, exacerbations of asthma and chronic obstructive pulmonary disease, or changes in testing, including forced expiratory volume, chest x-rays, and computed tomography scans.
- Outcomes (cardiovascular): measures of cardiovascular function, including blood pressure, heart rate, carotid intima-media thickness, and coronary artery calcifications, or changes in cardiovascular disease symptoms (clinical observation or self-reported).

### Objectives

We are conducting 2 systematic reviews to critically assess and synthesize available human studies on the respiratory and cardiovascular health effects of ENDS substitution for people who smoke. We will provide an even-handed assessment of the data, considering all health effects, both potentially adverse and beneficial. The primary goal is to provide clinicians with evidence on the health effects of ENDS substitution for people who smoke to inform their treatment recommendations and plans. The twin goals of the reviews include promoting health literacy in ENDS users with facts on the health effects of ENDS with a white paper drawn from the review. These 2 systematic reviews will be conducted with the living methodology to keep the information up to date and complete, as described in the following sections.

## Methods

### Overview

The protocol conforms to the PRISMA-P (Preferred Reporting Items for Systematic Reviews and Meta-analyses Protocols) requirements [30]; the completed checklist is provided in Multimedia Appendix 1. This protocol is registered with PROSPERO (CRD42021239094). Any deviations from this protocol will be reported in the reviews. These reviews are being conducted concurrently, so there is an overlap between their methodological procedures. Nevertheless, each review has unique testing data and differing effect modifications from participants’ smoking history that necessitate conducting separate reviews for respiratory and cardiovascular health effects.

These 2 reviews will be living systematic reviews. Living systematic reviews are a recent innovation for conducting systematic reviews that incorporate evidence from studies as they are published [31,32]. Living systematic reviews follow the established methods of conducting a systematic review and in addition perform searches and publish updated reviews at prespecified intervals. This enhancement overcomes the major issue of systematic reviews becoming outdated soon after publication [33].

The Cochrane guidelines [32] specify 3 conditions to justify conducting a living systematic review: (1) a lack of high-quality studies or certainty about them, (2) the priority of the evidence for decision making, and (3) emerging data that have a significant impact on the conclusions of previous reviews. Our review questions satisfy all of the above conditions. First, there are a relatively limited number of human studies on the health effects of ENDS, contributing to the uncertainty of their clinical impact [27,34,35]. Second, decisions by clinicians and policy makers are often based on beliefs that are not supported by the available studies [36-38]. Research evidence on the health risks of ENDS is a priority for decision making because ENDS could represent an excellent tobacco harm reduction opportunity [34] if their long-term risk reduction when compared with smoking would be demonstrated. Finally, new evidence is being published that is expected to impact existing knowledge. Indeed, the research output on ENDS has increased rapidly since 2018 [39,40]. Our preliminary search of articles on ENDS in PubMed retrieved 1332 articles published in 2019 and 1565 articles published in 2020 (search in Multimedia Appendix 1). The living systematic review format is clearly in order.

The review team comprised RO, the project leader, with extensive experience in conducting literature reviews and a substantial background in tobacco control, tobacco harm reduction, and ENDS. The research team comprised 4 fellows: MAQ, GRMLR, and DCO, each having literature review experience as the first author of a narrative review, and RWMV, who has substantial experience in literature reviews. MAQ and GRMLR are clinicians, and DCO has worked as a pharmacist. MAQ has a background in tobacco control and tobacco harm reduction.

The processes that will be conducted to create the baseline reviews are described in the following sections.

### Study Selection

Study designs selected for the reviews include randomized and nonrandomized controlled trials and clinical trials, prospective and retrospective cohort studies, and case-control studies. A gray literature search was performed. Database searches were performed separately for the cardiovascular and respiratory studies. Supplementary searches were conducted after the database search and after the full paper selection.

#### Gray Literature Search

A search for articles on ENDS not published in peer-reviewed journals was conducted on January 13, 2021, on the websites of 41 cardiovascular medical organizations and 53 respiratory medical organizations (Multimedia Appendix 1). No gray literature was found.

#### Database Search and Secondary Searches

The databases searched included Scopus, PubMed, and CENTRAL Cochrane Library.

The keywords used for the search included the following: ENDS keywords were *electronic cigarettes* and *e-cigarettes*. Vapor or vapor were not used as keywords, as these terms retrieve many chemical studies. Cardiovascular keywords were cardiovascular, heart, circulatory, arterial, and stroke. The respiratory keywords were lung, pulmonary, and respiratory.

The text fields searched were title and abstract in PubMed; title, abstract, and keywords in Scopus; and trials in Cochrane Library.

The search dates were from 2010 to January 31, 2021. The start date of 2010 is the date of publication of the first peer-reviewed research studies on ENDS.

The languages searched were English, French, Spanish, and studies in any language with an English abstract.

In compliance with PRISMA-P, an example of the search strategy is reported in the Multimedia Appendix 1.

The retrievals were entered into EndNote for bibliographic management. Paper retrieval duplicates were removed. There were 374 retrievals cardiovascular outcomes and 703 retrievals for respiratory outcomes.

The first exclusion of articles was performed on titles, and where a title was not sufficient for a determination, the abstract was reviewed. There were five categories of exclusion criteria. The first category was article types: editorials, commentaries, letters, news articles, issue introductions, and reviews. The second category was studies that were not peer reviewed, including conference abstracts, unpublished clinical trials, and preprint articles. The third category was articles without an English abstract (except for those in French or Spanish). The fourth category was studies exclusively on EVALI (e-cigarette or vaping product use–associated lung injury) because most EVALI cases were linked to black market tetrahydrocannabinol products not commercial ENDS [41]. The fifth category was the study design, including in vitro, inhalation toxicology, biomarker, animal, cross-sectional studies, and studies conducted exclusively with youth.

The exclusion process was conducted independently by 2 reviewers, and all discrepancies were resolved by discussion between the reviewers. One study in a nonincluded language was excluded. The number of studies after title and abstract exclusion was 43 cardiovascular outcomes studies and 23 respiratory outcomes studies.

As a supplementary search, the reviews discussed in the introduction [15,18-28] were searched independently by 2 reviewers for studies with the exclusion criteria above.

The second process of inclusion and exclusion was a full-paper review. There were 3 inclusion criteria. First, studies were limited to the research designs of randomized controlled trials, nonrandomized clinical studies, prospective or retrospective cohort studies, and case series. Second, a study was required to have either a comparator of participants who smoked combustible tobacco (cigarettes) or a before and after testing of participants who had substituted ENDS for smoking. Third, the study had to report outcome data on cardiovascular or respiratory function or diseases. All 3 aforementioned criteria had to be met for a study to be included.

The review team members were trained and supervised on the inclusion criteria on 5 studies. The inclusion and exclusion of studies was conducted independently by 2 reviewers, and discrepancies were resolved by a discussion. The reviewers achieved 95% (63/66) agreement on the inclusion and exclusion of studies. The project leader made the final decision on the studies that were questioned.

After this step, the reference lists of all included studies were reviewed for additional studies and were citation chased in Google Scholar. A list of studies excluded during the full-paper review is reported in the Multimedia Appendix 1.

The search processes yielded 27 cardiovascular outcomes studies and 19 respiratory outcomes studies that are listed in the Multimedia Appendix 1. The list of the included studies for each review will be sent to 2 medical experts for examination to ensure that no relevant studies have been missed. Any additional studies will be reported in the review.

### Data Extraction Process

The data extraction process is being conducted with a data extraction form. The reviewers were trained using calibration exercises to ensure consistency. Data are being extracted independently by 2 reviewers and will then cross-checked by the reviewers for accuracy and completeness. Any discrepancies in data extraction will be rectified by discussion. The data extraction items were drawn from inventories by JBI and the Cochrane Collaboration [42,43]. The data extraction form is provided in Multimedia Appendix 1. The categories include bibliographic details, population data, description of the intervention, respiratory or cardiovascular functioning or disease outcomes, data analysis, and study conclusions. If the published data are judged as insufficient or missing, the corresponding author will be sent an email with a request for additional details.

Two additional examinations of each study are being performed. The first is a check for internal discrepancies in the reporting of data [44] (Multimedia Appendix 1). The second is a comparison of the study as it was conducted with its protocol or clinical trial registration where one is available (form in Multimedia Appendix 1).

The completed data extraction forms will be submitted to the Systematic Review Data Repository at the Agency for Healthcare Research and Quality [45], an open access database.

Data from these studies will be reported in two ways. First, individual studies will be presented with a brief narrative description. Second, study tables will be constructed with items from the data extraction and quality assessments (see the following section).

### Quality Assessment and Risk of Bias

A quality assessment of each study is being conducted using the JBI quality assessment tool for its research design [46]. Reviewers were trained on the JBI quality assessment tools with an examination of their questions and a discussion of examples of quality issues. The quality assessment of the statistical analyses will be double-checked by a reviewer (RO or RWMV) with training in biomedical statistics.

Bias is being assessed with observations of biases listed in the Oxford Centre for Evidence-Based Medicine *Catalogue of Bias* [47] applicable to the study designs in the review. The review team prepared a set of prompting questions for each type of bias and was briefed on common examples. The checklist of biases is provided in Multimedia Appendix 1.

Quality assessment and risk of bias observations are being made concurrently with the data extraction process. Two reviewers are independently performing the quality and bias assessments. Discrepancies will be resolved through discussions between the reviewers. If a consensus is not reached, the final decision will be made by the project leader. No studies will be excluded based on their quality assessment or risk of bias observations. Studies not conforming to the JBI quality assessment items or with observations of biases will be reported in the study tables. These shortcomings will be specified in the data analysis and will be referenced in the discussion section of the reviews.

### Data Analysis and Synthesis

The synthesis will be a narrative synthesis. Owing to the heterogeneity of the study populations and outcome measurements, we anticipate that a meta-analysis will be inappropriate. If sufficient studies are identified as comparable either during the baseline review or for updated reviews, we will develop an additional protocol for a meta-analysis and will add it to the review.

The narrative syntheses will have four components. The first will be the findings organized by the study design. The second will be a summary of descriptive statistics for the participants and intervention characteristics. The third will group the findings based on the tests performed, physiological functions, and disease outcomes. The final synthesis will tally studies that have been found to demonstrate quality issues or biases.

Three subgroup analyses of testing and disease outcomes will be conducted for (1) concurrent users of cigarettes (dual users), (2) populations with prior respiratory or cardiovascular diseases, and (3) ENDS use of a duration of 1 year or longer.

Sensitivity analyses will be performed to explore the influence of the risk of bias on the findings. One will be to rerun the third analysis by excluding all studies assessed at a high risk of bias. A set of sensitivity analyses will be performed on groups of studies based on the following types of funding sources and author affiliations: ENDS or tobacco industry, pharmaceutical affiliations, and philanthropic or medical organization affiliations or funding. Publication bias will be assessed using a funnel plot for the test results and disease outcomes.

The certainty evidence for each reviews’ findings will be evaluated with the Grading of Recommendations Assessment, Development, and Evaluation framework [48,49].

### Update Plan

#### Updating the Study Search

A literature search will be conducted at 3-month intervals to retrieve newly published studies. Searches were conducted using the same databases and keywords as the baseline review. Newly published papers will be checked for studies that meet the inclusion criteria. The searches will apply appropriate date limiters to include only those records added to the database subsequent to the last search, allowing for indexing lag time [31,32]. Searches will be maintained in an EndNote library that will record all the search results over the lifetime of the review. Notes about each new search and the inclusion or exclusion decision for each study will be recorded. The newly included studies will be checked for references. The PRISMA (Preferred Reporting Items for Systematic Reviews and Meta-Analyses) diagram will be updated accordingly.

The first update search will be conducted on or shortly before April 30, 2021. The retrieved studies will be incorporated into the baseline review so that it will be updated when published.

The updating search can result in 3 scenarios: (1) no new studies are identified (highly unlikely), (2) new studies are retrieved but the evidence has no impact on the review’s findings, or (3) new studies are found with evidence that significantly impacts the review’s findings. This third scenario will trigger an immediate update of the review [31,32]. In evaluating the impact of new evidence, we will consider whether it causes a change in the Grading of Recommendations Assessment, Development, and Evaluation certainty rating or introduces previously unreported interventions, populations, serious adverse events, or other clinically meaningful findings [32,50]. Any of these conditions will trigger an updated review.

#### Updating the Review

Because a large volume of new studies is expected, we will update the review every 6 months if no studies prompt an earlier revision of the review. Narrative descriptions of the newly included studies and new study table listings were produced by merging them with the baseline review studies. All data analyses will be conducted using the procedures used for the baseline review. Conclusions and recommendations are revised to reflect the addition of new studies.

In addition, the search methods will be reviewed annually, or sooner if substantial changes occur that impact the search methodology, such as new search terms or sources. Possible changes to the frequency of the searches will be considered. We will verify that the scope of the review (ie, population, intervention, comparator, and outcomes components) is reflected in the inclusion of adequate thesaurus terms or changes to database search syntax. Other methodological aspects such as the use of technology enablers will also be reviewed annually.

One addition to the scope of this review has been made for the COVID-19 pandemic. As tobacco smoking may be a risk factor for more severe COVID-19 disease outcomes, we will include in the reviews any clinical studies on the impact of ENDS substitution for smoking on patients with COVID-19, particularly with regard to *long COVID*. No studies in this area have been retrieved from the initial search.

#### Transition Out of Living Mode

The review will be transitioned out of living mode if the research question no longer meets all the 3 criteria justifying the living approach. At that time, we will examine article-level metrics, knowledge translation activities, and the output of new research studies. A practical factor that could result in the end of the living mode is reaching the end of our funding in September 2023. After this period, new funding will be sought to maintain the living mode. If funding is not acquired, we will ask if at least two members of the review team are available as volunteers for 1 year of updates, followed by a final revision of the review. If both of these strategies fail, the review will be transitioned into a traditional systematic review for final publication at the end of the project.

## Results

The goal of these reviews is to assemble all the available human studies on the respiratory and cardiovascular effects of ENDS in people who smoke. Furthermore, the reviews will assess the quality and potential biases of the studies to foreground the best available evidence. The reviews will identify those studies that demonstrate reporting bias so that the misrepresentation of ENDS health effects can be addressed in the contentious debate around tobacco harm reduction. The living systematic review methodology will keep the evidence current and complete as opposed to a static systematic review that quickly becomes out-of-date due to the rapid pace of publication of ENDS studies.

As of March 11, 2021, the literature search has been completed except for the review of the study selection by experts, with 27 studies included in the cardiovascular outcomes review and 19 studies in the respiratory outcomes review. Training on data extraction, quality assessment, and bias assessment processes has been completed. The data extraction process has commenced. The target date for the completion of the reviews is July 2021.

## Discussion

Few protocols contain substantive planning for dissemination or knowledge translation activities beyond the publication of the review in a peer-reviewed journal and conference presentations. Of course, the reviews will be disseminated through these traditional avenues. This protocol has been published as a preprint on medRxiv. Pending acceptance, the project protocol will be published in the *Journal of Medical Internet Research Protocols*. The baseline and updated reviews will be published in a peer-reviewed journal that agrees to work with the living format for updated editions of the review. The abstracts for the reviews will be translated into as many languages as possible. Announcements of these publications will be sent out on social media platforms (Twitter, Facebook, etc).

In addition, we will write white papers to make the findings of the reviews accessible to clinicians and current or potential ENDS users. The reviews and white papers will be made available for downloading on a dedicated website. The reviews and white papers will be added as references to the relevant Wikipedia pages.

For clinicians, a white paper will spell out the treatment considerations of ENDS use drawn from both the cardiovascular and respiratory outcomes from the reviews. As most physicians and health care providers hold erroneous beliefs about the health effects of nicotine itself [51-53], the white paper will include a section on nicotine. The white paper will be translated into as many languages as possible and sent to medical associations, distributed at conferences, and published on a website for downloading.

For current and potential ENDS users, a white paper with infographics will explain the health effects found in the reviews. The public also hold misperceptions about the health effects of nicotine [11,54], so this white paper will have a section on nicotine. The white paper will be sent to the International Network of Nicotine Consumer Organizations, vapor product magazines, the Cochrane Consumer Network, Consumers United for Evidence-Based Healthcare, and patient advocacy organizations concerned with smoking-related diseases. We will explore producing short videos for YouTube and TEDx based on the white paper.

Our goal with these actions is to achieve a wide dissemination of the findings of the reviews to a wide audience. Standard review publication practices would fail to reach the stakeholders who will benefit from having the evidence presented in a format that is accessible and readily understood.

The terrible toll of death and disease from cigarette smoking calls for every effort to stem the tobacco epidemic. The substitution of ENDS for cigarettes is one way to potentially reduce the risks associated with smoking. Clinicians and their patients who smoke need to understand the benefits of substituting ENDS for cigarettes. They also need to be aware of the risks because “research toward uncovering the risks of e-cigarette use is aligned with optimizing harm reduction” [55]. Our living systematic reviews seek to highlight the best and most up-to-date evidence in this highly contentious and fast-moving field of research.

## Appendix

Multimedia Appendix 1 Supplementary materials.

## Abbreviations

ENDS: electronic nicotine delivery systems
EVALI: e-cigarette or vaping product use–associated lung injury
NASEM: National Academies of Sciences, Engineering, and Medicine
PRISMA: Preferred Reporting Items for Systematic Reviews and Meta-Analyses
PRISMA-P: Preferred Reporting Items for Systematic Reviews and Meta-analyses Protocols
